# Blastic plasmacytoid dendritic cell neoplasm with a history of cytopenia: A case report

**DOI:** 10.1002/dc.24463

**Published:** 2020-05-06

**Authors:** Miao Yan, Wensheng Wang, Xinan Cen, Lihong Wang, Yuhua Sun, Bingjie Wang, Jinping Ou, Lin Nong, Hanyun Ren, Ping Zhu, Mangju Wang

**Affiliations:** ^1^ Department of Hematology Peking University First Hospital Beijing China; ^2^ Department of Pathology Peking University First Hospital Beijing China

**Keywords:** Blastic plasmacytoid dendritic cell neoplasm, chronic myelomonocytic leukemia, histogenesis

## Abstract

Blastic plasmacytoid dendritic cell neoplasm (BPDCN) is a rare and aggressive hematologic malignancy arising from plasmacytoid dendritic cell precursors. The disease typically manifests in the skin, but it also evolves into a leukemic phase or can be complicated by other myeloid malignancies, especially myelomonocytic tumors. The association between these neoplasms is not fully elucidated. We report a case of BPDCN with a history of cytopenia that was supposed to be chronic myelomonocytic leukemia. The patient received intensive chemotherapy and achieved complete remission, but soon relapsed. The successive occurrence of myelomonocytic neoplasm and BPDCN is in accordance with the fact that they evolve from a common cell origin with a multilineage potential for myelomonocytic and plasmacytoid dendritic cell differentiation. This case may shed further light on the mystery of biology and the histogenesis of BPDCN.

## 1 INTRODUCTION

Blastic plasmacytoid dendritic cell neoplasm (BPDCN) is a rare disease characterized by malignant proliferation of blastic plasmacytoid dendritic cells, which are generally found positive for CD4, CD56, as well as antigens associated with plasmacytoid dendritic cells (pDCs), such as CD123, TCL1, BDCA2, and BDCA4.^1^ It is known that BPDCN is closely related to other myeloid neoplasms, which usually occur before the diagnosis of BPDCN or during the follow‐up stage.^1^, ^2^, ^3^ However, the biological relationship between these malignancies and BPDCN is not interpreted. In this study, we report a case of BPDCN with a preceding history of cytopenia, which was interpreted most likely as chronic myelomonocytic leukemia (CMML). The identification of such cases may shed new light on BPDCN biology and histogenesis.

## 2 CASE REPORT

A 67‐year‐old Chinese man, with a history of leucopenia and thrombocytopenia in the past 10 years, was admitted to our hospital for cutaneous lesions. The lesions appeared on the skins of the chest, back, and abdomen 5 months earlier, and have since spread to the face and legs. Physical examinations discovered multiple infiltrative plaques varying from light red to purple. Routine laboratory results revealed leukocyte count of 4.1 × 10^9^/L (4‐10 × 10^9^/L), platelet count of 54 × 10^9^/L (100‐300 × 10^9^/L), monocyte percentage of 34% (3.0%‐10.0%) and monocyte count of 1.4 × 10^9^/L (0.1‐0.6 × 10^9^/L).

**FIGURE 1:**
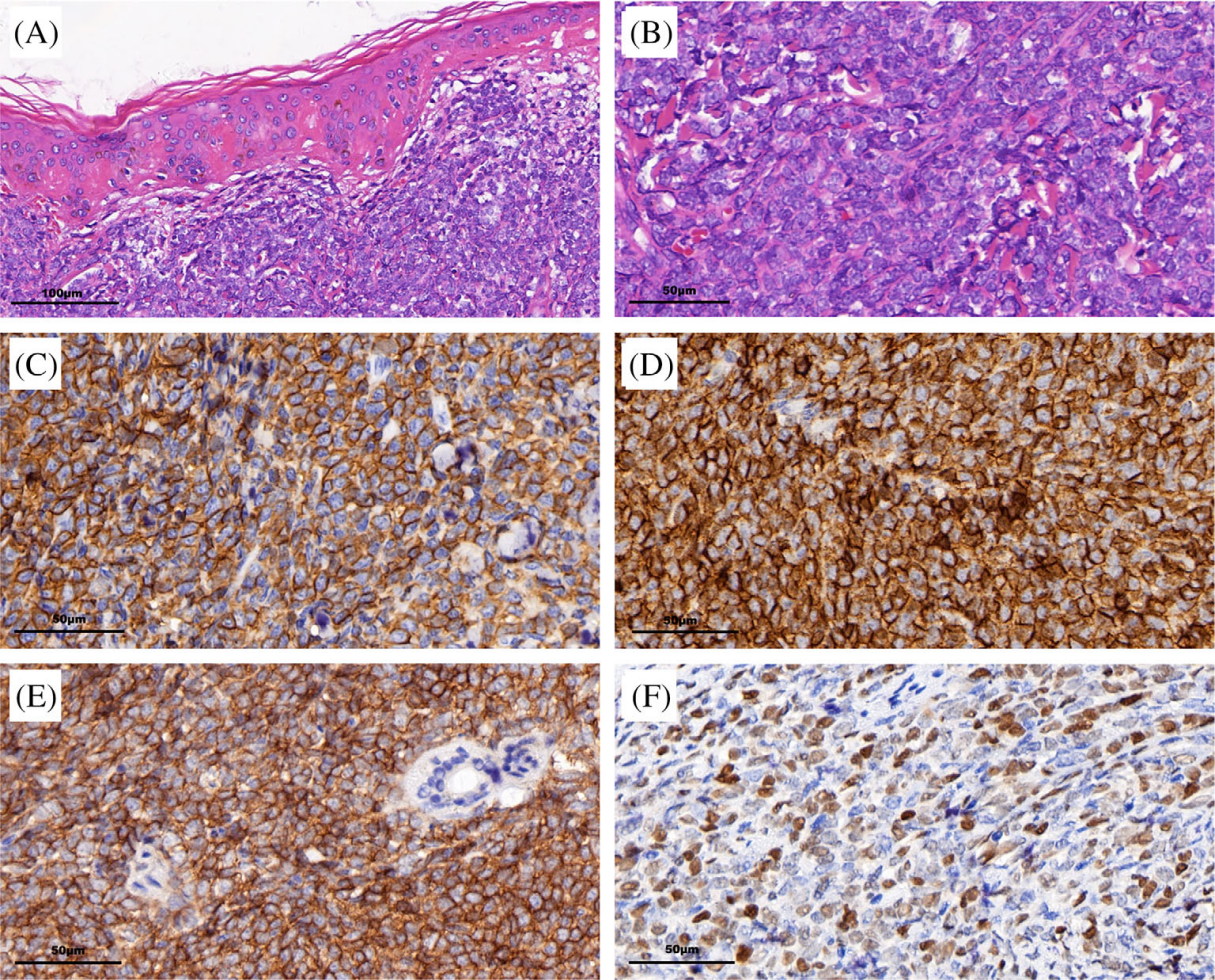
Histopathology of the skin lesion. A and B, Hematoxylin and eosin staining (A, ×200, B, ×400) showed infiltration of blastoid large cells in dermis and subcutaneous adipose tissue with irregular nuclei contours, fine granular chromatin, visible nucleolus, and several mitotic cells. C to F, Immuno‐histochemical staining (×400) revealed infiltrative cells positive for CD4, C, CD56, D, CD123, E, and TdT, F [Colour figure can be viewed at wileyonlinelibrary.com]

The histological examination of a biopsy specimen taken from a plaque on the chest demonstrated the presence of dermis and subcutaneous adipose tissue infiltration of blastoid large cells, but no epidermis involvement (Figure 1). Immunohistochemistry (IHC) analysis showed that cells were positive for CD4, CD56, CD123, CD43, and TdT (Figure 1), and negative for CD3, CD20, MPO, CD68, Granzyme B, CD34, CD117, CD2, CD7, and CD138. The Ki67 labeling index of the tumor cells was 60%.

**FIGURE 2:**
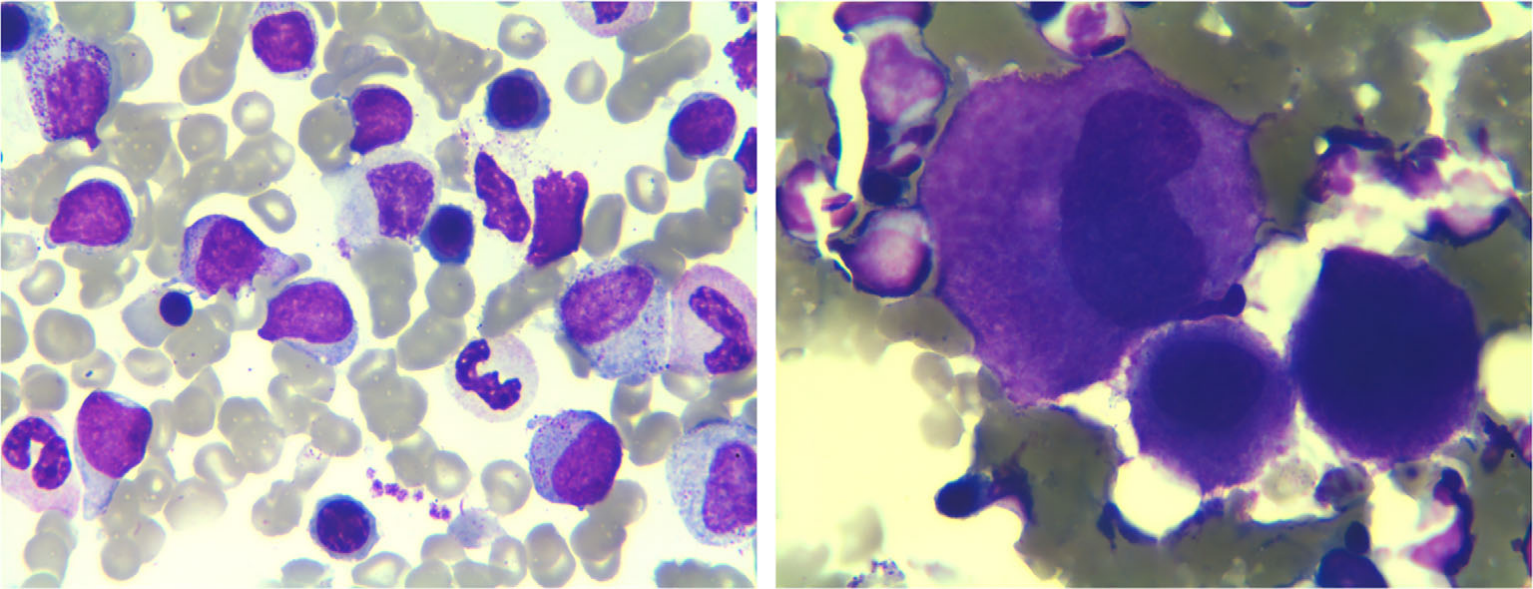
Images of bone marrow smear. Wright‐Giemsa staining showed myeloid hyperplasia, dysplastic hematopoiesis, and blastic cells with loose chromatin, 0 to 3 nucleolus and scant gray‐blue cytoplasm [Colour figure can be viewed at wileyonlinelibrary.com]

**FIGURE 3:**
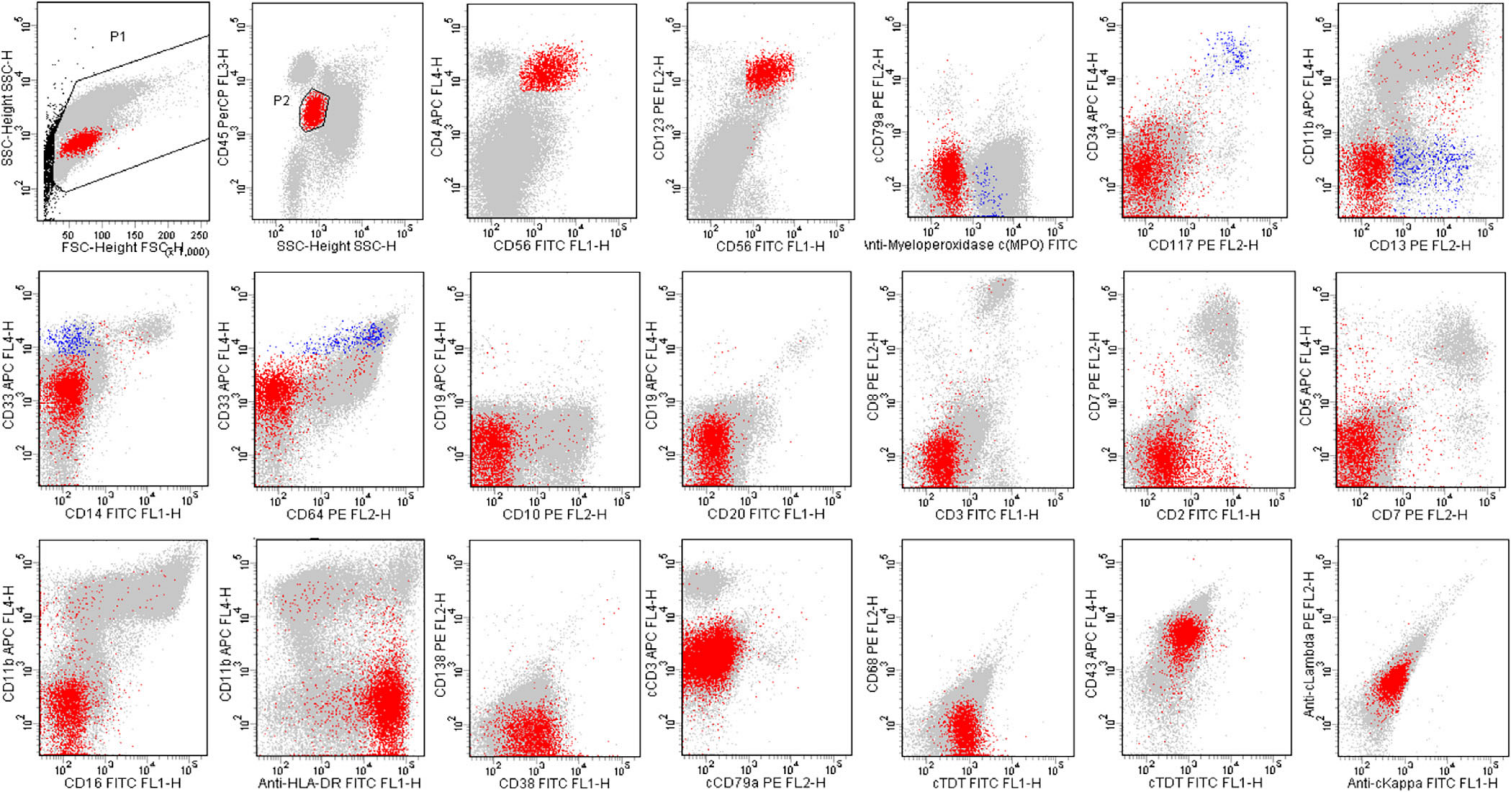
Flow cytometric analysis of bone marrow. The 3.5% of the nucleated cell gate (red signals) was characterized as CD123+, CD4+, CD56+, CD34‐, CD38+, cTdT+, cCD79a−, cCD3−, cMPO−, CD117−, CD33−, CD13−, CD11b−, CD64−, CD14−, CD10−, CD19−, CD20−, CD43+, CD68−, CD2−, CD3−, CD5−, CD7−, CD8−, CD16−, cKappa− and cLamda−, and 0.28% of the nucleated cell gate (blue signals) was positive for cMPO, CD34, CD117, CD13, CD33, and CD64 [Colour figure can be viewed at wileyonlinelibrary.com]

The results of bone marrow smear demonstrated the presence of an extremely active myeloid hyperplasia and dysplastic hematopoiesis (Figure 2). Flow cytometric analysis of the bone marrow revealed two groups of phenotypically abnormal cells. One group exhibited the immunophenotype consistent with the IHC features of the cutaneous lesions (Figure 3, red signals), and the other group displayed characteristics of blastic myeloid cells (Figure 3, blue signals). One percentage of the peripheral blood nucleated cells was also detected to be positive for CD123, CD4, and CD56. Mutation of CALR (Exon9) L367fs*46 was found positive in the detection for myeloproliferative neoplasm associated genes. However, detection for MDS‐related genes was not conducted on the samples.

**FIGURE 4:**
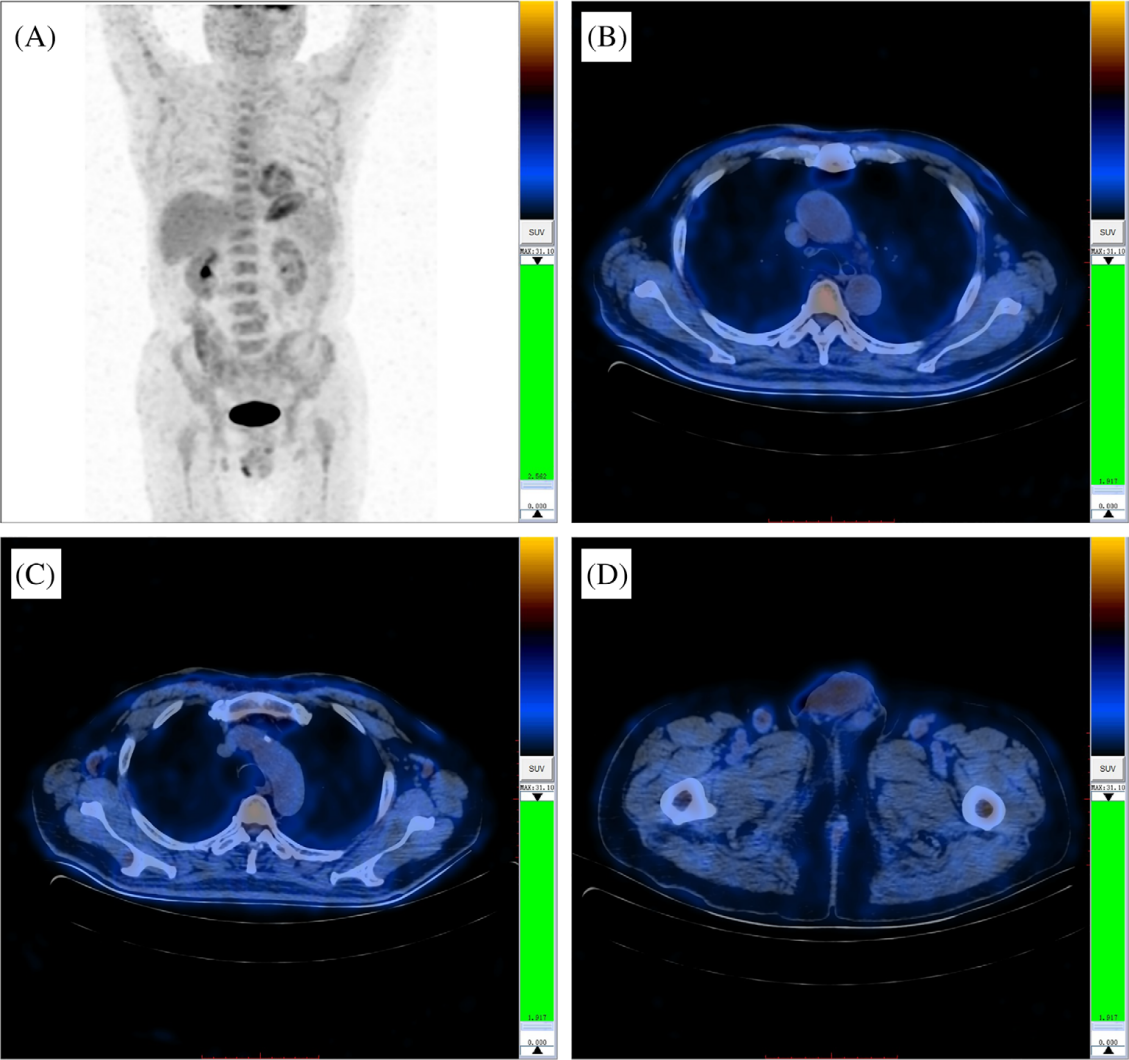
Images of FDG PET‐CT. A, PET maximum intensity projection (MIP) image suggested multiple mild FDG‐avid cutaneous lesions and involvement of lymph nodes and trunk bone. B, PET‐CT fusion image showed thickened and mild FDG‐avid cutaneous lesions on the chest. C and D, PET‐CT fusion images revealed involvement of bilateral axillary and inguinal lymph nodes [Colour figure can be viewed at wileyonlinelibrary.com]

**FIGURE 5:**
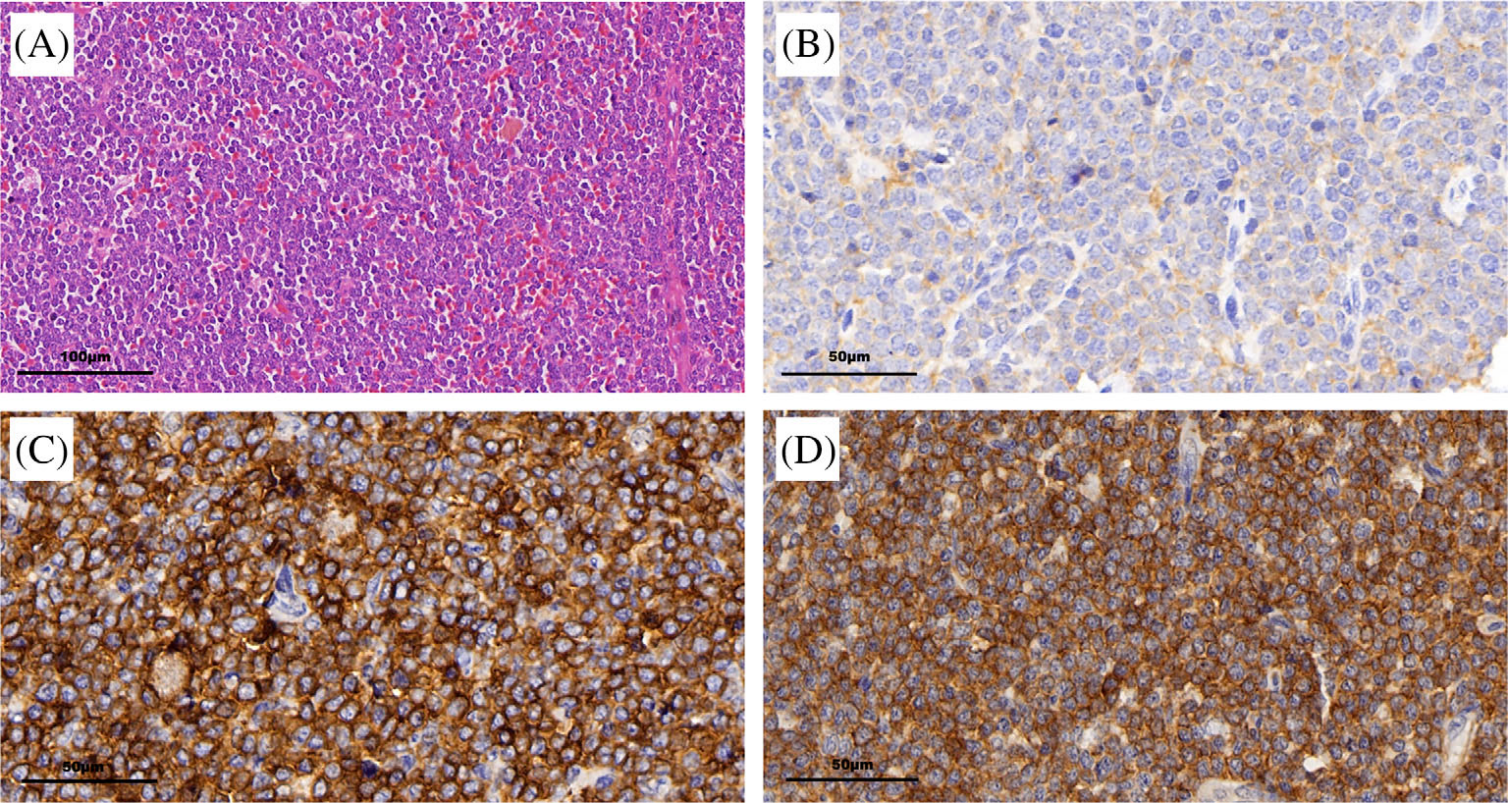
Histopathology of the inguinal lymph node biopsy. A, Hematoxylin and eosin staining (×200) showed that the normal lymph node structure was destructed and replaced by abnormal diffuse infiltration of large naive lymphoid hematopoietic tumor cells. B to D, Immuno‐histochemical staining (×400) revealed the cells positive for CD4, B, CD56, C, and CD123, D [Colour figure can be viewed at wileyonlinelibrary.com]

Subsequently, the patient underwent an F^18^‐fluorodeoxyglucose positron emission tomography‐computed tomography (FDG PET‐CT), which revealed the involvement of the skin, bone marrow, and multiple lymph nodes (Figure 4). IHC analysis of the inguinal lymph node biopsy reported CD3−, CD20−, CD68−, MPO−, CD56+++, CD4+, CD123+++, TdT +, CD34−, and Ki67 40% (Figure 5).

The patient was diagnosed as BPDCN with skin, lymph nodes, bone marrow, and peripheral blood involvement, as well as a speculated history of CMML. He received VDCLP‐regimen chemotherapy (vindesine, idarubicin, cyclophosphamide, pegaspargase, and prednisone). During the treatment, the cutaneous lesions faded. The patient acquired complete remission and hematopoietic stem cell transplantation was scheduled. However, his subsequent cerebral infarction canceled the treatment and any further aggressive therapeutic procedures. As expected, the disease recurred, and the patient died 1 year after diagnosis.

## 3 DISCUSSION

BPDCN is a rare entity with a specific immunophenotype. In this case, the blast cells of the skin, bone marrow, and lymph nodes biopsies were characterized by the co‐expression of CD4, CD56, and CD123 in the absence of specific myeloid or lymphoid lineage markers. In accordance with previous observations,^4^, ^5^ TdT and CD43 were positively expressed. However, flow cytometry suggested that the patient had a simultaneous abnormal blastic myeloid group in the bone marrow.

It was recognized that the normal counterpart of BPDCN resides in pDCs, which are one of the two functionally distinct subsets of dendritic cells. Up to 20% of BPDCN patients have a preceding history or complication of myeloid hematologic malignancies, including myelodysplastic syndrome, chronic myeloid leukemia, and CMML.^1^, ^2^, ^3^, ^6^ BPDCN often progresses to a leukemic phase, which leads to a poorer prognosis.^7^ The relationship between BPDCN and other myeloid malignancies is not clearly elucidated. It has been speculated that BPDCN and other myeloid neoplasms may share a common clonal origin, and the developmental plasticity of precursors may potentiate the rise of both malignancies.^3^, ^6^ BPDCN and CMML were reported to significantly overlap in the spectra of gene mutations, including TET2, ASXL1, SRSF2, and NRAS.^8^ Moreover, myeloid tumors display several features that are found in pDC‐related tumors, and CD56 expression in myeloid leukemias, is associated with skin involvement and monocytic differentiation.^1^, ^6^

In our case, the patient presented with a preceding history of cytopenia, but no immune‐phenotypic and cytogenetic data from recent years were available. It was not until he developed skin damage that blast cells in the bone marrow and peripheral blood were discovered. Bone marrow flow cytometry analysis displayed two groups of immuno‐phenotypically abnormal cells, indicating distinct tumor clones. One group was consistent with the skin biopsy, reflecting BPDCN bone marrow involvement, the other group expressed myeloid precursor markers, and even though their percentage was low, an overly concentrated distribution suggested their abnormality. Particularly, the patient presented thrombocytopenia and monocytosis with monocytes representing more than 10% and an absolute count that was greater than 1 × 10^9^/L in the peripheral blood. The bone marrow smear showed myeloid hyperplasia and obvious dysplasia. Mutation of CALR, a typical gene mutation in myeloproliferative disorders, was also detected. Taken together, the patient may have a history of CMML. We believe the successive occurrence of BPDCN and CMML was related rather than accidental. As previously reported, various hematologic malignancies evolve from clonal hematopoiesis, presumably as a consequence of additional mutations.^9^ It is reasonable to suppose that different malignant clones may derive from a common cell origin, and that different genes evolvement drove respective differentiation. For our case, the patient was assumed to acquire other gene mutations following CMML during the process of aging, which promoted the occurrence of BPDCN. To clarify the histological association between BPDCN and myelogenous neoplasms, it is necessary to sequence the embryonic or the two‐separated tumor cells to identify common or driving mutational evidence in future studies. We believe that this is a valuable case that provided clues to our understanding of the potential relationship between the two tumors and that this case enriched our clinical experience on the occurrence mode of this rare disease.

In conclusion, we report a case of BPDCN with a history of cytopenia. We believe that the association of BPDCN and various myeloid disorders, especially myeloid‐monocytic neoplasms, is more than coincidental. Our case provides further support to a common origin between BPDCN and myelogenous neoplasms.

## CONFLICT OF INTEREST

The authors declare that they have no conflict of interest.

## AUTHOR CONTRIBUTIONS

Miao Yan, Wensheng Wang, Xinan Cen, Hanyun Ren, Ping Zhu, Yuhua Sun, and Bingjie Wang were the treating physicians. Lihong Wang and Jinping Ou analyzed the flow cytometry data. Lin Nong analyzed and interpreted the pathological data. Miao Yan and Mangju Wang designed the study and drafted the manuscript. All authors read and approved the final manuscript.

## ETHICS STATEMENT

All the examinations and treatments in this case report were clinical routines which were necessary for accurate diagnosis and improved outcome. Informed consent for all diagnostic measures and treatment options was obtained from the patient and his family.

## CONSENT FOR PUBLICATION

The patient has passed away and written informed consent was obtained from the patient's wife for the publication of this case report and accompanying images.
